# The gut microbiota and host immunity synergistically orchestrate colonization resistance

**DOI:** 10.1080/19490976.2025.2611545

**Published:** 2026-01-03

**Authors:** Na Li, Xiaohuan Guo

**Affiliations:** a Institute for Immunology, Tsinghua University, Beijing, People's Republic of China; b School of Basic Medical Sciences, Tsinghua Medicine, Tsinghua University, Beijing, People's Republic of China; c Beijing Key Laboratory of Immunological Research of Allergy (LIRA), Tsinghua University, Beijing, People's Republic of China; d State Key Laboratory of Molecular Oncology, School of Basic Medical Sciences, Tsinghua University, Beijing, People's Republic of China; e SXMU-Tsinghua Collaborative Innovation Center for Frontier Medicine, Shanxi Medical University, Taiyuan, Shanxi, People's Republic of China

**Keywords:** Colonization resistance, gut microbiota, host immunity, IL-22, epithelial glycosylation, host-microbiota interaction

## Abstract

Colonization resistance is a fundamental host defense mechanism that relies on the synergistic interaction between the gut microbiota and the host immune system to prevent enteric pathogen colonization and infection. This review synthesizes current knowledge of the multifaceted mechanisms governing colonization resistance against intestinal pathogens. We examine how commensal microbes directly suppress pathogens through niche and nutrient competition, contact-dependent inhibition, and the production of antimicrobial compounds and metabolites. From the host perspective, we outline the essential roles of gut barriers, innate and adaptive immunity, and antimicrobial peptides in maintaining microbiota homeostasis while selectively restricting pathogen expansion. We also emphasize the role of IL-22 signaling and its regulation of epithelial glycosylation, which modulates nutrient availability and shapes microbial competitiveness. Finally, we discuss key challenges and future research directions in colonization resistance and related translational research, with the goal of informing novel strategies to prevent and treat intestinal infections and inflammatory diseases.

## Introduction

The gut microbiota forms a complex ecological network with the host, and helps us with nutrient metabolism, immune system development, and the barrier maintenance. One of its most fundamental roles is colonization resistance, which is the capacity of the indigenous microbiota, together with host defenses, to prevent the establishment and overgrowth of invading pathogens.^1-4^ The concept of colonization resistance dates back to the 1950s, when Marjorie Bohnhoff proposed that streptomycin-induced dysbiosis could increase the host susceptibility to pathogens, while certain beneficial members of the microbiota could outcompete harmful ones.^5^ Subsequent studies using antibiotics and germ-free (GF) animals demonstrated that the absence of commensal bacteria increased susceptibility to pathogens such as *Salmonella enterica* and *Clostridioides difficile (C. difficile),*^6-10^ highlighting the essential role of microbiota in preventing pathogen colonization. Colonization resistance relies on two interdependent components: the gut microbiota and the host immune system. The microbiota provides direct protection by occupying ecological niches, competing for nutrients, producing bacteriocins and engaging in contact-dependent inhibition, as well as generating bioactive metabolites that modulate both microbial ecology and host physiology.^3^ The host contributes through physical and chemical mucosal barriers as well as innate and adaptive immune responses.^11^ Disruption of colonization resistance has profound clinical consequences. Perturbations such as broad-spectrum antibiotic use, dietary shifts, or immunosuppression can destabilize microbial communities and impair barrier function, thereby permitting opportunistic overgrowth, such as *C. difficile*, or increasing susceptibility to enteric pathogens.^5^^,^^12-15^ Understanding the mechanisms that sustain colonization resistance is essential for designing interventions to prevent and treat infections, and for restoring host-microbiota homeostasis after disturbance. This review summarizes the molecular and cellular mechanisms underlying CR from both host and microbiome perspectives, emphasizing their bidirectional interactions and exploring clinical implications for the prevention and treatment of enteric infections.

### The role of the gut microbiota in colonization resistance

**Figure 1.**
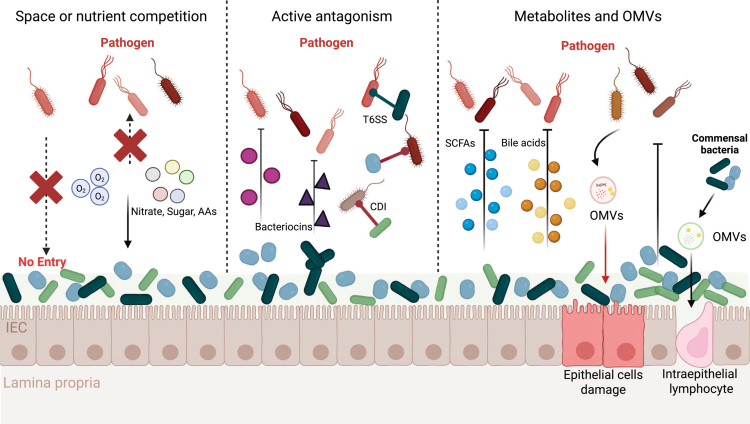
Direct mechanisms of microbiota-mediated colonization resistance. Commensals directly prevent pathogen colonization and invasion. (1) Resident commensal bacteria occupy the ecological space, preventing pathogens from establishing a niche and invading the intestinal epithelial cells; and commensal bacteria stave out pathogens by consuming the essential nutrients (oxygen, nitrate, sugar and amino acids). (2) Commensal bacteria suppress and eliminate pathogen through multiple direct mechanisms, such as secreting bacteriocins, inhibiting growth via contact-dependent inhibition, and deploying the T6SS. (3) Commensal bacteria suppress pathogen through metabolites (SCFAs, bile acids); while pathogen-derived OMVs promote epithelia cell damage, commensal OMVs enhance colonization resistance by supporting beneficial flora and intraepithelial lymphocyte.

The gut microbiota plays a central role in colonization resistance by directly limiting invading pathogens through multiple ecological and molecular mechanisms. These include competition for physical niches and nutritional resources, production of antimicrobial factors, contact-dependent inhibition, and the generation of bioactive metabolites that modulate pathogen fitness and virulence.^16-18^ Together, these activities establish a dynamic and resilient ecosystem that protects the host from colonization by exogenous microorganisms (Figure 1).

### Direct competition for spatial niches and nutrients in colonization resistance

Colonization resistance is fundamentally based on the ecological competition between commensals and invading pathogens. The intestinal environment is densely populated, with resident microbes occupying key spatial niches along the mucus layer and epithelial surface.^19^ By colonizing these sites, commensals physically block pathogens from accessing the adhesion sites necessary for successful colonization.^20^ Spatial competition plays a pivotal role in colonization resistance. For instance, although both commensal *Escherichia coli (E. coli)* and pathogenic *Salmonella enterica serovar Typhimurium (S. Typhimurium)* require nitrate for growth. *E. coli* can exploit niches created by *S. Typhimurium* virulence factors and inhibit pathogen expansion through nitrate competition.^21^ Similarly, commensal *Enterobacteriaceae* compete with *Salmonella enteritidis* for oxygen, another resource critical for pathogen proliferation.^22^ *Clostridia* confer colonization resistance via PPAR-*γ*-mediated hypoxia, restricting facultative anaerobes *Candiada albicans (C. albicans)* and *E. coli*, antibiotic disruption elevates oxygen and promotes pathogen expansion.^23^ Conversely, certain non-antibiotic drugs have been shown to inhibit commensal growth, disrupt spatial exclusion, and enhance *S. Typhimurium* access to newly available niches.^24^

In addition to spatial exclusion, competition for essential nutrients is a key mechanism limiting pathogen expansion. Different bacterial species specialize in metabolizing specific substrates, creating a tightly regulated metabolic landscape. For instance, *Bacteroidetes* degrade complex polysaccharides, thereby limiting carbohydrate availability to enteric pathogens such as *S. Typhimurium* and pathogenic *E. coli*. Commensal bacteria *Klebsiella oxytoca* protect against multidrug-resistant *Klebsiella pneumoniae (K. pneumoniae)* by actively competing for specific *β*-glucosides, a mechanism that requires the casA protein and cooperative interactions with other commensals.^25^ *Klebsiella michiganensis* also outcompetes pathogenic *E. coli* for critical nutrients. Notably supplementation with galactitol, a sugar preferentially utilized by *E. coli*, abolishes this protection, highlighting nutrient competition as the dominant mechanism.^26^

Pathogens have evolved strategies to overcome these competitive pressures. *S. Typhimurium* uses its virulence factors to gain access to glucose through a combination of aerobic respiration and mixed acid fermentation, enabling it to break colonization resistance.^27^ In more complex microbial communities, commensal *E. coli* alone is insufficient to prevent invasion. However, in mice colonized with a defined 12-strain community (OMM12)-a gnotobiotic mouse model harboring a minimal, well-defined microbiota-*E. coli* can deplete galactitol, a substrate otherwise exploited by *S. Typhimurium*, thereby reducing pathogen colonization.^28^ Conversely, galactitol supplementation can promote co-existence of multiple *S. Typhimurium* strains and facilitate plasmid transfer, demonstrating how nutrient dynamics shape both colonization resistance and the spread of resistance genes.^29^ Similarly, the recently identified commensal *Dubosiella newyorkensis* (L8) mediates exercise-induced colonization resistance against methicillin-resistant *Staphylococcus aureus* (MRSA) by depleting fucose, an essential carbon source for MRSA growth and pathogenicity.^30^

Nutrient competition extends beyond carbohydrates to include amino acids, which also play critical roles in colonization resistance. The gut microbiota can deplete local amino acids, restricting pathogen growth. In response, pathogens activate biosynthetic pathways to compensate. For example, a synthetic fecal microbiota transplantation (sFMT) inhibited *C. difficile* colonization by reconstructing microbial networks that specifically competed for proline, whereas supplementation with certain amino acids reversed this inhibition.^31^ Dietary protein similarly influences colonization resistance. Exclusion of dietary protein suppresses antibiotic-enhanced *Salmonella* colonization, indicating that both diet and microbiota jointly regulate amino acid availability.^32^ Furthermore, *C. albicans* can release millimolar levels of arginine into the intestinal lumen, which in turn induces the type III secretion system in *S. Typhimurium*, enhancing epithelial invasion while dampening host inflammatory responses.^33^

Collectively, these studies highlight that direct competition for space and nutrients is a fundamental mechanism of colonization resistance.

### Bacteriocins in the colonization resistance

Many commensal bacteria strengthen colonization resistance through the production of bacteriocins and other antimicrobial peptides, which directly inhibit pathogen growth.^34^ These compounds are typically narrow-spectrum, selectively targeting closely related bacterial strains. By eliminating competitors, they provide a fitness advantage to the producing species while promoting stability and resilience within the gut microbiota.

A notable example is a commensal consortium containing *Blautia producta* BPscsk, which secretes a lantibiotic structurally similar to nisin A. This lantibiotic effectively suppresses the growth of vancomycin-resistant *Enterococcus faecium* (VRE). While both BPscsk and *Lactococcus lactis* inhibit VRE in vitro, only BPscsk can establish stable intestinal colonization and significantly reduce VRE burden in vivo.^35^ Similarly, bacteriocin-driven competition has been linked to lineage replacement within *Enterococcus* populations. Plasmid-encoded bacteriocins, such as bacteriocin 21 carried on the conjugative plasmid pPD1 in *Enterococcus faecalis* (*E. faecalis*)*,* enable the producing strain to displace resident enterococci and dominate the intestinal niche.^36^

Beyond enterococci, other commensals utilize similar strategies to exclude pathogens. For instance, *Lactiplantibacillus paraplantarum* produces plantaricin RX-8 to attenuate *Listeria monocytogenes* colonization and intestinal inflammatory response.^37^ Likewise, members of the *Klebsiella oxytoca* complex secrete enterotoxins such as tilimycin and tilivalline, which contribute to colonization resistance against pathogens like *S. Typhimurium* through both direct toxin-mediated inhibition and competition for key nutrients.^38^ Importantly, nutrient availability modulates the efficacy of these interactions, emphasizing the dynamic interplay between bacteriocin activity and metabolic competition in maintaining colonization resistance.

Together, bacteriocins represent a powerful mechanism by which commensal microbes directly suppress pathogenic populations, thereby safeguarding intestinal homeostasis and protecting the host from infection.

### Contact-dependent inhibition systems in colonization resistance

Contact-dependent inhibition constitutes a potent, spatially constrained mechanism of colonization resistance in which effector proteins are delivered from one bacterium directly into neighboring cells via specialized secretion apparatuses—most notably the Type VI secretion system (T6SS).^39^ In densely populated habitats such as the colon, where intercellular proximity is high, T6SS-mediated toxin injection enables commensals to arrest growth or kill closely competing strains, thereby securing and stabilizing local niches.^40^ Members of the *Bacteroidales* exemplify this strategy and several gut *Bacteroidales* deploy T6SS effectors to antagonize conspecifics and heterologous competitors, resulting in contact-dependent growth inhibition.^41^ Similarly, *K. pneumoniae*, an opportunistic pathogen and frequent gut colonizer, relies on a functional T6SS to eliminate specific gram-negative competitors, particularly *Betaproteobacteria*, thereby promoting its persistence in the gut. T6SS expression in *K. pneumoniae* is tightly regulated and induced under gut-like conditions, highlighting how this system contributes to its ecological success and pathogenic potential.^42^ Complementary in vivo work illustrates the ecological impact of such direct antagonism: a commensal consortium including *Blautia producta* and *Clostridium bolteae* restores colonization resistance to VRE and clears VRE from murine intestines, with *Blautia producta* showing direct inhibitory activity against VRE.^43^

Importantly, contact-dependent inhibition operates in concert with host-mediated spatial control of the microbiota. For example, nociceptor neurons limit the density of microfold (M) cells in Peyer’s patch follicle-associated epithelium, thereby reducing entry points for *Salmonella* invasion; downstream of this axis, nociceptors sustain levels of *segmented filamentous bacteria* (SFB) on ileal villi and follicle-associated epithelium, and SFB in turn mediates resistance to *S. Typhimurium.*^44^ Thus, host mechanisms that sculpt microbial biogeography can potentiate or constrain contact-dependent antagonism by determining which microbes occupy the immediate physical niches required for contact-dependent inhibition.

In summary, contact-dependent inhibition, principally via T6SS and related systems, provides a direct, contact-dependent form of microbial interference that, together with host-driven spatial organization, plays a critical role in preventing pathogen establishment and maintaining intestinal homeostasis.

### Microbial metabolites as mediators of colonization resistance

In addition to direct microbial antagonism, gut microbiota-derived metabolites play a pivotal role in shaping colonization resistance by modulating both microbial competition and host physiology. These metabolites function as chemical signals and nutrient sources, influencing pathogen growth, virulence expression, and the overall ecological balance within the gut.

Short-chain fatty acids (SCFAs), such as acetate, propionate, and butyrate, are among the most extensively studied metabolites.^45^ Produced through the fermentation of dietary fibers by commensal bacteria, SCFAs enhance intestinal barrier integrity and regulate luminal oxygen levels.^46^ For instance, butyrate promotes epithelial hypoxia via PPAR-*γ* activation, thereby creating an anaerobic environment that limits the expansion of facultative anaerobes like *S. Typhimurium,*^23^ while it also directly suppresses *C. difficile* by inhibiting its butyrate metabolism and virulence gene expression.^47^ Propionate exerts direct inhibitory effects by disrupting the intracellular pH of *S. Typhimurium*, thereby suppressing pathogen growth.^48^ However, *S. Typhimurium* can counteract this inhibition by metabolizing propionate as a carbon source for anaerobic respiration, a strategy dependent on commensal propionate-producing species such as *Bacteroides thetaiotaomicron* (*B. thetaiotaomicron*).^49^ This highlights a dynamic metabolic interplay in which the same metabolite can either reinforce or undermine CR depending on the microbial context.

Bile acid metabolism represents another central axis of metabolite-mediated colonization resistance. Commensal bacteria, such as *Clostridium scindens*, transform primary bile acids into secondary bile acids through enzymatic activities including 7α-dehydroxylation and bile salt hydrolase (BSH) activity.^50^ Secondary bile acids, particularly deoxycholic acid (DCA), inhibit the germination and vegetative growth of *C. difficile* spores, thereby serving as a key barrier to infection.^51^ Perturbations that reduce bile acid diversity, such as antibiotic treatment or extreme dietary interventions like very-low-calorie diets, are associated with increased susceptibility to *C. difficile* colonization. Recent studies further reveal that distinct commensal BSH repertoires modulate the bile acid pool, influencing resistance not only to *C. difficile* but also to other enteric pathogens such as *Vibrio cholerae*, whose virulence gene expression depends on bile salts like taurocholate.^52^^,^^53^ Sleep deprivation has also been shown to disrupt secondary bile acid homeostasis-identified through metabolomic profiling, which captures global small-molecule metabolic changes-lowering DCA levels and consequently impairing colonization resistance against pathogens such as MRSA and pathogenic *E. coli*.^54-56^

Collectively, these findings underscore that microbiota-derived metabolites function at the interface of microbial ecology and host defense. By regulating nutrient availability, metabolic cross-feeding, and signaling pathways, these compounds create a dynamic and context-dependent landscape that can either promote pathogen clearance or, when disrupted, facilitate pathogen expansion and infection.

### Outer membrane vesicles in colonization resistance

Outer membrane vesicles (OMVs) are nanoscale, bilayered particles released by Garm-negative bacteria that encapsulate diverse biomolecules, including protein, toxin, lipids, nucleic acids, polysaccharide-modifying enzymes and various metabolites.^55^ By carrying this multifaceted cargo, OMVs play critical roles in various physiological processes-such as intracellular and intercellular communication, quorum sensing, horizontal gene transfer, interbacterial killing, toxin delivery, polysaccharide degradation, and stress responses-positioning them as key mediators of microbial ecology.^56^

Recent studies show that OMVs contribute significantly to colonization resistance through multifaceted interactions between pathogens, commensals, and the host. Pathogenic species, including enterotoxigenic *E. coli* (ETEC), *Vibrio cholerae*, and *S. Typhimurium,* exploit OMVs to transport virulence factors and toxins into host cells, inducing cytotoxicity and inflammatory responses that facilitate infection.^57-59^ In contrast, OMVs derived from gut commensals represent an emerging area of interest. Many resident bacteria continuously shed OMVs containing bioactive components that serve as essential messengers in host-microbiota communication. For example, OMVs from *Akkermansia muciniphila (A. muciniphila)* can selectively fuse with specific gut bacteria, promoting the expansion of beneficial taxa, reducing opportunistic pathogens, and restoring microbial richness and diversity under dysbiotic conditions.^60^ Similarly, Bacteroides species increase OMVs production under environmental stress. These commensal OMVs possess immunogenicity and can trigger an IFN-MHC-II-intraepithelial lymphocyte axis, thereby enhancing mucosal defense against intestinal infection.^61^

Together, these findings underscore OMVs as dynamic modulators of the gut ecosystem, simultaneously shaping microbial community structure and fortifying host resistance to pathogen colonization.

### The role of host immunity in colonization resistance

Host immunity contributes fundamentally to colonization resistance by mounting coordinated, multi-layered defenses that act synergistically with the microbiota to prevent pathogen colonization. Ranging from physical and chemical barriers to sophisticated innate and adaptive responses, this integrated system not only restricts pathogen access but also maintains immune homeostasis toward the resident microbial community.^62^

### Mucus as the first line of defense in the intestinal tract

**Figure 2.**
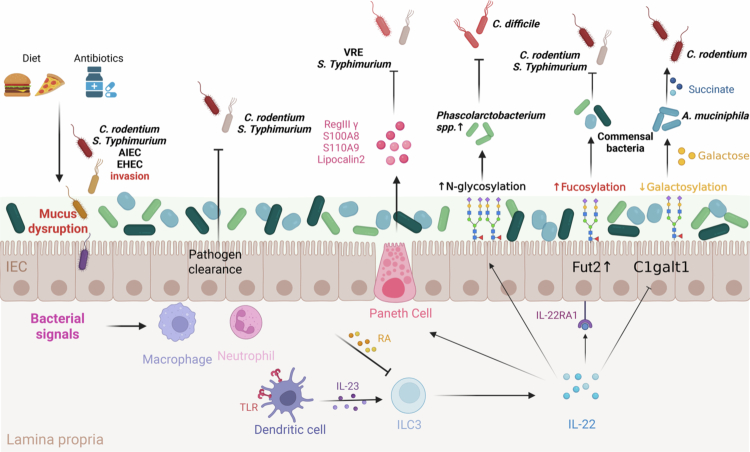
Innate immune mechanisms in regulating pathogen colonization resistance. The innate immune system orchestrates multiple pathways to defend against pathogen colonization. (1) Dietary factors and antibiotic exposure disrupt the mucus barrier, enabling invasion by multiple pathogenic bacteria (*C. rodentium*, *S. Typhimurium*, AIEC, EHEC). (2) Microbe-derived signals recruit macrophages and neutrophils to participate in pathogen clearance (*C. rodentium*, *S. Typhimurium*). (3) Activation of TLR signaling in dendritic cells induces IL-23 production, which stimulates ILC3 to secrete IL-22; RA further modulates excessive IL-22 responses. IL-22 mediates colonization resistance primarily by inducing antimicrobial peptides and by regulating diverse intestinal glycosylation pathways (e.g., *N*-glycosylation, fucosylation, galatosylation). IL-22-dependent enhancement of *N*-glycosylation promotes the commensal bacterium to resist lethal *C. difficile* infection. IL-22-driven fucosylation supports symbiotic bacteria in limiting pathogen colonization (e.g., *C. rodentium*, *S. Typhiumurium*). Additionally, IL-22 suppresses galactosylation, thereby regulating *A. muciniphila*-mediated succinate production and constraining *C. rodentium* infection.

The intestinal mucus layer forms a critical interface that physically separates the gut microbiota and the host epithelium. Primarily secreted by goblet cells and architected around the highly glycosylated mucin MUC2,^19^ it mediates essential host-microbiota interactions through two major mechanisms (Figure 2). First, the microbiota regulates the production and maintenance of the mucus layer. For instance, GF mice exhibit an attached small intestinal mucus layer. Upon colonization with microbiota, the mucus gradually normalizes and becomes detached over the course of 5–6 weeks, demonstrating that microbial presence is essential for proper mucus organization.^63^ Second, mucus itself provides binding sites and nutrients that support microbiota growth. The glycans within mucins can be directly utilized by certain bacteria. For example, *A. muciniphila* and *B. thetaiotaomicron* are specialized in binding to and degrading mucin-derived glycans, thereby accessing a nutrient source that is not influenced by host digestive enzymes.^63-65^ By metabolizing distinct glycans, these bacteria occupy different metabolic niches within the gut ecosystem.

The integrity and composition of the mucus layer are closely linked to colonization resistance against enteric pathogens. GF mice, which possess a thinner mucus layer, are more susceptible to severe infections.^63^ Similarly, mucus impairment induced by diet perturbation or MUC2 deletion leads to increased host susceptibility to pathogens such as *Citrobacter rodentium (C. rodentium)*, resulting in higher bacterial loads and more severe disease outcomes.^66^^,^^67^ In streptomycin-induced intestinal dysbiosis, the cecal mucus layer becomes disrupted, allowing *S. Typhimurium* to swim more easily through the mucus and directly attach to intestinal epithelial cells (IECs) to initiate infection.^68^

Interestingly, mucus overproduction can have protective effects. For instance, an increase in mucus abundance supports the growth of *A. muciniphila*, which in turn helps resist infection and intestinal inflammation caused by adherent-invasive *E. coli* (AIEC).^69^ Moreover, mucus can sustain the expression of virulence factors in *Bacteroides fragilis* and Enterohemorrhagic *E. coli (EHEC)*, thereby maintaining host–microbiota homeostasis under steady-state conditions.^70^^,^^71^

In summary, mucus functions as a critical first line of defense by physically segregating pathogens from IECs, thereby preventing direct bacterial contact and epithelial invasion. At the same time, it shapes the composition and activity of the commensal microbiota, promoting colonization resistance and maintaining intestinal homeostasis.

### Innate immunity in colonization resistance

Innate immunity plays a pivotal role in reinforcing colonization resistance through rapid, nonspecific defense mechanisms that limit pathogen invasion and expansion. A key component of this response involves pattern recognition receptors (PRRs), such as Toll-like receptors (TLRs), which sense microbial signals and trigger protective pathways.^72^ Early studies demonstrated that TLR signaling is essential for regulating infection susceptibility. For example, gut-derived lipopolysaccharide (LPS) activates the TLR4 pathway, leading to the production of the antimicrobial peptide RegIIIγ, which is critical for preventing VRE colonization.^12^ Consistently, MYD88-deficient mice exhibit compromised intestinal barrier function and significantly elevated pathogen burdens following *C. rodentium* infection, highlighting the essential role of TLR-MYD88 signaling in maintaining colonization resistance.^15^

Beyond these canonical innate pathways, neutrophils and macrophages have emerged as central effectors in shaping colonization resistance through direct pathogen clearance and modulation of microbiota–host interactions. During *S. Typhimurium* infection, rapid neutrophil recruitment to the intestinal lumen limits pathogen expansion while simultaneously enhancing the activity of commensal *E. coli* strains such as Nissle 1917. Neutrophil-derived reactive oxygen species potentiate siderophore-bound toxin activity, thereby promoting competitive displacement of *S. Typhimurium.*^73^ Similarly, disruption of intestinal epithelial cell autophagy, as seen with *Atg7* deficiency, leads to a microbiota shift enriched in Gram-positive bacteria that drives early neutrophil and phagocyte infiltration. This response accelerates clearance of *C. rodentium*, mitigates epithelial hyperplasia, and subsequently promotes protective Th17 and Treg immune responses.^74^ In parallel, macrophages serve as key regulators of tissue homeostasis and barrier defense. Fibroblast-derived CSF1 maintains macrophage populations within specialized intestinal niches, where they directly interact with pathogens and coordinate immune responses. Loss of fibroblast CSF1 disrupts this macrophage network, resulting in impaired local defense and systemic dissemination of *S. Typhimurium.*^75^ Collectively, these findings highlight that efficient colonization resistance relies on the dynamic recruitment and functional specialization of neutrophils and macrophages, which act in concert with epithelial and microbiota-derived signals to contain enteric pathogens.

Among innate immune mediators, interleukin-22 (IL-22) plays a central role in defending against enteric infections and preserving intestinal epithelial homeostasis.^76^ IL-22 is primarily secreted by group 3 innate lymphoid cells (ILC3s) and acts directly on IECs.^77^ A major function of IL-22 is the regulation of antimicrobial peptides (AMPs) production, which is crucial for controlling pathogenic colonization.^78^ For instance, stimulation with the TLR7 agonist R848 activates CD11c⁺ dendritic cells (DCs) to produce IL-23, which in turn induces a burst of IL-22 secretion by ILCs. This cascade promotes RegIIIγ expression, thereby restoring colonization resistance against VRE.^79^ Interestingly, one study demonstrated that *Clostridia*-driven IEC-intrinsic retinoic acid (RA) synthesis can suppress excessive IL-22 production and associated antimicrobial responses, leading to reduced expression of AMPs such as RegIIIγ, S100A8, and S100A9. This change thereby promotes the expansion of commensal bacteria belongs to phylum Firmicutes, which ultimately prevents the expansion of pathogenic *S. Typhimurium* in the gut.^80^ IL-22 also regulates other AMPs, including lipocalin-2, which limits bacterial access to iron. Resistance to lipocalin-2-mediated iron sequestration is a specific adaptation that enables *S. Typhimurium* to thrive in the inflamed gut.^81^

Beyond regulating AMPs, IL-22 further promotes colonization resistance by modulating epithelial glycosylation. The ILC3–IL-22–glycosylation axis has emerged as a key mediator of this host-microbiota synergy. Initial research established intestinal epithelial fucosylation, catalyzed by fucosyltransferase 2 (Fut2), as a crucial element of host-microbiota mutualism. ILC3s mediate commensal bacteria-induced epithelial through the production of IL-22, and perturbation of this pathway increases host susceptibility to *S. Typhimurium* infection, underscoring its essential protective function.^82^ In addition, systemic infection-particularly through TLR activation-stimulates dendritic cells to produce IL-23, which activates ILC3s to secrete IL-22 and promotes the production of fucosylated proteins. This response supports commensal growth to resist *C. rodentium* invasion.^83^ Moreover, IL-22-mediated fucosylation plays a critical role in controlling opportunistic pathogen *E. faecalis*. Loss of IL-22 receptor (IL-22RA1) results in diminished epithelial fucosylation, which promotes epithelial translocation of pathogenic *E. faecalis* and consequently increases susceptibility to lethal *C. rodentium* infection.^84^ IL-22-regulated glycosylation also influences metabolic niche competition. In human microbiota-associated mice, IL-22 dependent *N*-glycosylation supports succinate-consuming commensals, reducing luminal succinate and constraining *C. difficile* growth. Impaired IL-22 signaling in ulcerative colitis elevates succinate levels and increases susceptibility to *C. difficile.*^85^

Our recent work has expanded this framework by linking ILC3s, intestinal galactosylation, and microbial metabolism. ILC3 deficiency increased epithelial galactosylation, facilitating overgrowth of the mucin-degrader *A. muciniphila*. Elevated *A. muciniphila*, identified through 16S rRNA sequencing-a technique that profiles microbial communities based on ribosomal gene markers -and its metabolite succinate, whose role in promoting *C. rodentium* virulence was demonstrated by bacterial genetics through targeted deletion of the succinate transporter *DcuB* in *C. rodentium*, together enhanced the expression of *C. rodentium* virulence genes, exacerbating infection. This reveals that ILC3-derived IL-22 controls broader glycosylation patterns that shape microbial community structure and pathogen behavior.^77^ Collectively, these studies delineate a model wherein the ILC3–IL-22 axis acts as a central hub linking host glycosylation, microbial ecology, and pathogen control. By regulating epithelial glycosylation, IL-22 shapes nutrient availability and competitive dynamics, stabilizing commensals and suppressing opportunistic pathogens. Disruption of this pathway predisposes the host to infections, underscoring its importance and revealing potential therapeutic strategies to restore CR by targeting IL-22-mediated mucosal regulation or glycan-dependent interactions.

In summary, innate immunity contributes to colonization resistance through multiple, interconnected pathways, including pathogen sensing, AMP production, and epithelial glycosylation (Figure 2). By coordinating these responses, innate immune mechanisms maintain intestinal homeostasis and provide a critical defense against enteric pathogen colonization.

### Adaptive immunity in colonization resistance

Adaptive immunity, both humoral and T cell–mediated immunity, plays a crucial role in enhancing colonization resistance by delivering antigen-specific and long-lasting protection that complements innate immune defenses.

A key effector of mucosal immunity, secretory IgA (sIgA), is produced by lamina propria plasma cells and transported across the epithelium into the intestinal lumen.^86^ By binding microbial antigens, sIgA inhibits pathogen adhesion to intestinal epithelial cells and neutralizes toxins without provoking significant inflammation, thereby helping to maintain a balanced host–microbiota interface. The integrity of this IgA-mediated barrier is highly dependent on early-life microbial maturation. Restricting microbiome maturation during weaning diminishes IgA induction and compromises colonization resistance, resulting in heightened susceptibility to Salmonella infection.^87^ Consistently, it is identified *Tomasiella immunophila r*educes intestinal sIgA levels through immunoglobulin-degrading proteases, thereby exacerbating vulnerability to *S. Typhimurium* and *C. albincans.*^88^ Neuroimmune circuits also contribute to IgA regulation, as enteric serotonergic neurons promote IgA^+^ B-cell differentiation through HTR7 signaling, enhancing mucosal defense against orally acquired *S. Typhimurium* infection.^89^ Mechanistically, sIgA can directly inhibit pathogen virulence. For instance, sIgA targeting *Shigella* impedes its type III secretion system, limiting epithelial invasion.^86^ Moreover, maternal sIgA and IgG responses can provide passive protection to offspring. Oral infection of female mice with *C. rodentium* stimulates the production of pathogen-specific sIgA and IgG antibodies that are transferred via breast milk. These antibodies, particularly IgG, recognize key virulence factors—such as the adhesin Intimin and the T3SS filament EspA—encoded within the locus of enterocyte effacement pathogenicity island, effectively protecting neonates against attaching-and-effacing pathogens.^90^

Beyond humoral immunity, T cell–mediated mechanisms are equally vital for colonization resistance. Th17 cells, through cytokines like IL-17 and IL-22, reinforce epithelial barrier function and stimulate antimicrobial peptide production, thereby restricting pathogen colonization.^91^ The importance of this axis is further highlighted by metabolic regulation within Th17 cells. Inhibition of glutathione synthesis markedly reduces IL-22 production by Th17 cells, thus impairing bacterial clearance and tissue repair and consequently worsening *C. rodentium* infection.^91^ Th17-dependent protection is also evident in other contexts. For example, colonization with *C. albicans* protects mice from lethal *C. difficile* infection.^92^^,^^93^ Pre-colonized mice show elevated IL-17A expression in infected tissues upon *C. difficile* challenge, and exogenous IL-17A administration alone can confer protection in the absence of *C. albicans*, underscoring the direct role of this cytokine in colonization resistance. Similar Th17-dependent protection is observed in other models. Female mice, for instance, clear MRSA from the gut more effectively than males, a difference attributed to microbiota-dependent expansion of IL-17A⁺ CD4⁺ Th17 cells and enhanced neutrophil recruitment.^93^ Additional T cell subsets contribute to barrier defense. During Salmonella infection, γδ intraepithelial lymphocytes undergo epithelial cell-dependent metabolic and migratory reprogramming that enhances their ability to counter intestinal infection.^94^ Regulatory T cells also play a nonredundant role: diphtheria toxin-mediated depletion of Tregs in Foxp3 (DTR) mice impairs *C. rodentium* clearance, exacerbates weight loss, and increases systemic dissemination.^95^ Type 2 immunity also contributes: in *Nod2*-deficient mice, helminth infection suppresses intestinal inflammation by inhibiting colonization of pro-inflammatory *Bacteroides* species. This protection is mediated by IL-13⁺ CD4⁺ T cells, which foster a protective microbiota enriched in Clostridiales.^96^

Commensal microbiota can also drive IFNγ-dependent protective responses. The transfer of a restricted consortium of cultivable commensals from resistant mice to susceptible hosts significantly reduced *S. Typhimurium* tissue colonization and disease severity. This effect required microbiota-enhanced IFNγ production, as IFNγ-deficient mice did not display protection. Following transfer, both innate immune cells and CD4⁺ T cells expanded and expressed high levels of IFNγ, indicating a cooperative role of innate and adaptive pathways in microbiota-mediated colonization resistance.^97^

**Figure 3.**
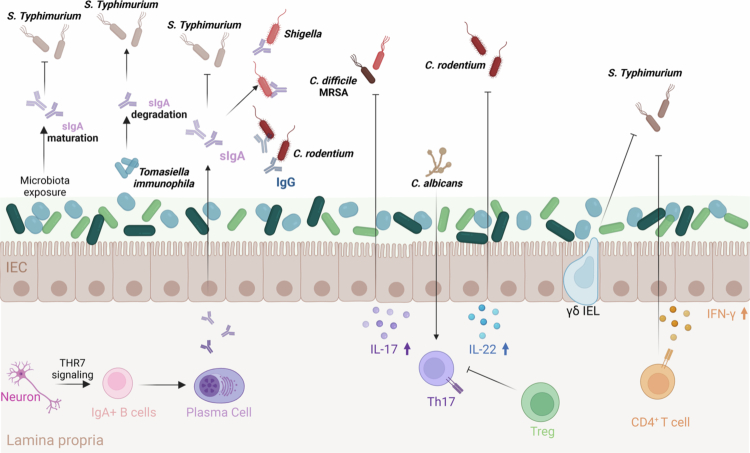
Adaptive immune mechanisms in regulating pathogen colonization resistance. The adaptive immune system provides specific defense against pathogen through humoral and cellular response. (1) Humoral immunity. Early-life exposure to commensal bacteria the maturation of sIgA, which enhances resistance to pathogenic colonization (*S. Typhimurium)*. *Thomasiella immunnouphila* promote sIgA degradation that enhances host susceptibility to *S. Typhimurium* infection. Intestinal neurons, through HTR7 signaling, drive the differentiation of IgA^+^ B cells into plasma cells, increasing sIgA production to against *S. Typhimurium* invasion. sIgA can also directly bind *Shigella* toxin to neutralize its activity, while IgG is essential for clearing *C. rodentium* infection. (2) C. albicans-induced Th17 responses promote IL-17 production to control *C. difficile* and MRSA infection, and additionally provide IL-22 to induce antimicrobial peptides and facilitate tissue repair during *C. rodentium* infection. Treg cells fine-ture Th17 activity to maintain balanced and effective colonization resistance. Intraepithelial lymphocytes and CD4^+^ T cells-derived IFN-*γ* further contribute to host defense against enteric *S. Typhimurium* infection.

In summary, adaptive immunity contributes to colonization resistance through pathogen-specific mechanisms involving both humoral and cellular responses. By producing targeted antibodies such as sIgA and IgG, as well as activating T cell subsets including Th17 and type 2 T helper cells, the adaptive immune system prevents pathogen adherence, enhances barrier integrity, and promotes the maintenance of a protective commensal community (Figure 3). These coordinated responses are essential for sustaining intestinal homeostasis and protecting the host from enteric pathogen colonization.

### The development of microbiota-based clinical therapies in infection

The maintenance of gut microbiota homeostasis is central to host resistance against pathogenic infections, driving significant interest in therapeutic strategies that remodel the intestinal microbial ecosystem. Fecal microbiota transplantation (FMT) represents a whole-microbiota intervention. The U.S. FDA has approved FMT for the treatment of recurrent *C. difficile* infection (rCDI), a condition strongly associated with antibiotic-induced dysbiosis.^98^ Clinical trials report that FMT achieves cure rates as high as 90% in rCDI.^99^ Beyond infectious diseases, accumulating evidence indicates that FMT may also serve as a potential therapeutic modality for inflammatory bowel disease, metabolic disorders, cancer and even neurological conditions.^100-103^

Despite its therapeutic promise, FMT remains a complex and heterogenous intervention with incompletely understood mechanisms of action. Several clinical challenges remain. Safety concerns are a major limitation: post-FMT complications such as gastrointestinal discomfort, sepsis, metabolic disturbances (weight gain) and exacerbation of ulcerative colitis have been reported.^98^^,^^104^ Potential risks include transmission of pathogens, mismatch of region-specific microbial communities, errors during transplantation procedures, and long-term, largely unknown consequences. These long-term risks may involve the transfer of predispositions to chronic diseases such as metabolic disorders, autoimmune diseases, neurological conditions, or even tumorigenesis.

These uncertainties have motivated the development of more precise, controllable microbiome interventions. Precision microbiome therapy aims to replace the crude transfer of whole fecal communities with defined, rigorously selected strains, rationally designed consortia, or functional microbial products tailored to correct specific dysbiosis. Significant preclinical progress has been made in precision therapy for *C. difficile*. For example, mixed *Lactobaciilus* probiotic formulations have been shown to reduced recurrence rates of *C. difficile*^105^; probiotics Enterococcus faecalis produce bacteriocin to inhibit toxigenic *C. difficile*^106^; and targeted anti-toxin approaches, such as gut microbial derived secondary bile acids, can inhibit toxin activity of *C. difficile.*^107^

In FMT, not only gut bacteria are transferred, but the viral component is also transmitted. The gut virome is predominantly composed of bacteriophages-viruses that specifically target bacteria-which are being explored clinically as a potential therapeutic strategy against pathogenetic bacterial infection.^108^ Bacteriophages are increasingly recognized as important contributors to colonization resistance, both by shaping microbial community structure and by selectively suppressing pathogenic bacteria. Beyond their ecological roles, phages hold significant clinical promise as targeted modulators of the gut microbiota. Unlike broad-spectrum antibiotics, phages exhibit high specificity, enabling precise elimination of pathogens such as *C. difficile*, antibiotic-resistant *K. pneumoniae*, and invasive *Salmonella* without disrupting beneficial commensals.^109-112^

Evidence from FMT further underscores the importance of phage in gut ecosystem restoration. A meta-analysis of 23 FMT studies that reconstructed and functionally annotated donor and recipient viromes show that patients with recurrent C. difficile infection experienced increased phage diversity following FMT. Notably, temperate phages successfully engraft and carry auxiliary metabolic genes that help regulate gut metabolic homeostasis and enhance resistance to infection.^113^ These findings demonstrate that FMT can remodel host metabolic functions through virome transfer, highlighting the virome’s previously underappreciated role in driving microbiome-mediated therapeutic effects.

Engineered or naturally occurring phages can also enhance colonization resistance by promoting niche stabilization, facilitating the growth of protective taxa, and modulating microbial metabolic outputs that support mucosal immunity.^114^ Advances in synthetic biology have accelerated the development of programmable phages capable of delivering antimicrobial payloads or re-sensitizing resistant strains to antibiotics. Early preclinical models and small-scale clinical studies demonstrate favorable safety profiles, minimal off-target effects, and durable pathogen suppression.^115^^,^^116^ Moreover, phage cocktails, phage-derived endolysins, and microbiome-informed personalized phage therapies are emerging as scalable strategies to prevent or treat recurrent and antibiotic-refractory infections.^117^

Microbiome-based therapeutic hold considerable promise, yet several major challenges must be addressed before broad clinical implementation. These include the design of individualized microbial formulations, comprehensive long-term safety evaluation, and reducing costs while improving standardization and controllability.

### Conclusion and future perspectives

Colonization resistance represents a fundamental defense mechanism resulting from the coordinated interactions between the host and the gut microbiota. It plays a central role in maintaining intestinal homeostasis and preventing the invasion and overgrowth of exogenous pathogens. This review has summarized the molecular and cellular mechanisms underlying colonization resistance, highlighting the contributions of both the gut microbiota and host immunity. The gut microbiota directly limits pathogen expansion through spatial niche occupation, nutrient competition, production of bacteriocins, and contact-dependent inhibition. Additionally, microbiota-derived metabolites, such as SCFAs and secondary bile acids, indirectly modulate the host barrier and microbial ecology to strengthen colonization resistance. OMVs derived from pathogen destroy host cells to promote infection, whereas OMVs produced by commensal bacteria strengthen host immune response to control pathogen infection. From the host perspective, multiple layers of defense, including the mucus barrier, antimicrobial peptides, innate immune sensing, and adaptive immune effectors, work together to maintain a balanced relationship with commensal microbes while restricting pathogenic colonization. In particular, IL-22 released by ILC3s regulates epithelial glycosylation to sustain colonization resistance. Collectively, colonization resistance emerges as a dynamic and multilayered process shaped by continuous cross-talk between the host and its microbiota.

Despite significant progress, several challenges remain. Clinically, translating mechanistic insights into therapeutic interventions is a priority. FMT and defined microbial consortia (sFMT) have shown promising efficacy in conditions such as *C. difficile* infection.^118^ However, these approaches face limitations including compositional instability, inter-individual variability, and concerns about long-term safety. Future research should focus on developing more precise and personalized strategies that integrate microbiota restoration with targeted metabolic interventions, selective probiotic consortia, or immune-modulating agents. Moreover, therapeutic modulation of host mechanisms, such as epithelial glycosylation and antimicrobial peptide production, represents an attractive avenue for enhancing mucosal defense. Interventions targeting key microbial metabolic pathways, including SCFA and bile acid metabolism, may also provide novel strategies for reinforcing colonization resistance and preventing infection recurrence.

Current studies largely focus on individual species or isolated mechanisms, yet the spatiotemporal dynamics of the host–microbiota ecosystem remain poorly defined. A key unanswered question is how immune pathways and microbial communities coordinate across distinct intestinal niches during pathogen invasion or therapeutic perturbation. For example, it remains unclear whether localized epithelial responses, such as IL-22–driven glycosylation, produce niche-specific microbial shifts that propagate along the gut. Another pressing issue is to identify the molecular mediators of cross-kingdom signaling, including how fungi, protozoa, archaea, and bacteriophages modulate bacterial competition and host immunity. Testing whether specific fungal metabolites or phage-encoded auxiliary genes directly influence CR represents a tractable experimental direction. Addressing these questions will require spatially resolved, longitudinal analyzes to determine which microbial functions—not just taxa—predict successful colonization resistance and how these functions change in real time under pathological or therapeutic conditions. Beyond bacteria, the gut harbors diverse viruses and fungi that remain understudied in the context of colonization resistance. Fungi such as *C. albicans* have been shown to modulate colonization resistance indirectly by influencing host immune responses, including IL-17 production,^119^ yet their overall contributions are only beginning to be understood. Similarly, the gut virome, especially bacteriophages, may shape bacterial communities and metabolic networks, thereby indirectly influencing colonization resistance. Future research should expand to include the virome and mycobiome, investigating their interactions with bacteria and the host immune system. Such studies will provide a more comprehensive understanding of the intestinal ecosystem and may uncover novel therapeutic targets.

In conclusion, colonization resistance represents a highly integrated and dynamic system involving microbiota, host immunity, and their metabolic interactions. Future research should bridge fundamental mechanistic studies with clinical applications, leveraging systems biology and precision medicine approaches. By strategically modulating both microbial communities and host defense pathways, it may be possible to develop innovative strategies for preventing and treating infectious diseases, inflammatory bowel disorders, and other conditions linked to disrupted intestinal homeostasis.

## Abbreviation

AMPs: Antimicrobial peptides
BSH: Bile salt hyfrolase
DCA: Deoxycholic acid
DCs: Dendritic cells
FMT: Fecal microbiota transplantation
Fut2: Fucosyltransferase 2
GF: Germ-free
IECs: Intestinal epithelial cells
ILC3s: Group 3 innate lymphoid cells
LPS: Lipopolysaccharide
M cells: Microfold cells
PRRs: Pattern recognition receptors
RA: Retinoic acid
SCFAs: Short-chain fatty acids
sFMT: Synthetic fecal microbiota transplantation
sIgA: Secretory IgA
TLRs: Toll-like receptors
T6SS: Type VI secretion system
AIEC: Adherent-invasive *E. coli*
*A. muciniphila*: *Akkermansia* *muciniphila*
*B. thetaiotaomicron*: *Bacteroides thetaiotaomicron*
*C. albicans*: *Candida albicans*
*C. difficile*: *Clostridioides difficile*
*E. coli*: *Escherichia coli*
*E. faecalis*: *Enterococcus faecalis*
EHEC: Enterohemorrhagic *E. coli*
MRSA: Methicillin-resistant *Staphylococcus aureus*
*K. pneumoniae*: *Klebsiella pneumoniae*
rCDI: Recurrent *C. difficile* infection
SFB: *Segmented filamentous bacteria*
*S. Typhimurium*: *Salmonella Typhimurium*
VRE: Vancomycin-resistant *Enterococcus faecium*
